# Mechanistic Insights Into Surfactant‐Regulated and Carboxylate‐Mediated Uniform Zinc Plating

**DOI:** 10.1002/cssc.70945

**Published:** 2026-08-03

**Authors:** Joachim Gerd Christian Hering, Erlendur Jónsson, Daniel Schröder

**Affiliations:** ^1^ Institute of Energy and Process Systems Engineering (InES) TU Braunschweig Braunschweig Germany

**Keywords:** aqueous zinc‐ion battery, electrode interface, electrolyte additive, zinc anode

## Abstract

Uniform zinc deposition and dendrite suppression are crucial objectives to enable high‐performance aqueous Zn‐ion batteries. In this study, a millimolar blend of sodium dodecylbenzenesulfonate (SDBS) and ethylenediaminetetraacetic acid (EDTA) achieves kinetic control over Zn plating in a mildly acidic electrolyte. Collectively, overpotential measurements, distribution of relaxation times analysis, and in situ Raman spectroscopy reveal a persistent coadsorption of SDBS and EDTA on the zinc electrode—slowing down interfacial charge–transfer kinetics and thus maintaining a constant Zn^2+^ surface concentration. This shift from diffusion control to interfacial kinetics fosters homogeneous metal growth and effectively suppresses dendrite formation. Comparative experiments with polyethylene glycol confirm that the surfactant’s efficacy derives from its ability to modulate plating kinetics rather than its molecular identity. Screening of trans‐1,2‐diaminocyclohexane‐N,N,N′,N′‐tetraacetic acid, L‐histidine, and zinc acetate further uncovers that freely available carboxyl (COO^−^) groups, instead of chelation strength, underpin extended cycle life. These mechanistic insights establish a general design principle for zinc metal anode‐containing battery systems: synergistic integration of a kinetic surfactant barrier with free COOH moieties to achieve prolonged cycling stability and uniform zinc deposition.

## Graphical abstract

Dendrite‐free zinc metal anodes are achieved by kinetic control of aqueous Zn plating using millimolar sodium dodecylbenzenesulfonate (SDBS) and ethylenediaminetetraacetic acid (EDTA) in a mildly acidic electrolyte. Persistent coadsorption slows interfacial charge–transfer, maintains Zn^2+^ surface concentration, and shifts growth from diffusion to interface kinetics. Free COO^–^ groups, rather than chelation strength, extend cycle life.

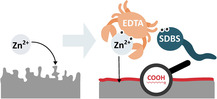

## 1 Introduction

The demand for sustainable and efficient energy storage solutions has led to considerable research into various battery technologies, one of which is zinc‐ion batteries (ZIBs). ZIBs represent a promising alternative to conventional lithium‐ion batteries for specialized applications, owing to their inherent benefits, such as the widespread availability of zinc resources, environmental sustainability, and high intrinsic safety [1]. Nevertheless, the practical implementation of ZIBs still faces significant challenges, including the formation of zinc dendrites, hydrogen evolution during cycling and rest periods, limited cycle life, and slow reaction kinetics [1]. It is imperative to address these challenges to fully harness the potential of ZIBs across a broad spectrum of applications, from medical on‐body devices to large‐scale energy storage systems [1, 2].

Since the development of the first zinc‐manganese dioxide batteries by Leclanché in 1860, primary zinc‐manganese dioxide batteries with alkaline electrolytes have achieved commercial success [3]. However, issues, such as the zinc anode passivation, complicate their use as rechargeable batteries [4]. In 1986, Yamamoto and Shoji introduced a battery with a neutral zinc sulfate electrolyte, which prevented passivation and enabled recharging [5]. In 2012, Xu et al. reported on a rechargeable battery that intercalates zinc in the manganese dioxide cathode, termed a zinc‐ion battery [6].

For the zinc anode of a ZIB, different modifications of metallic zinc or zinc alloys can be utilized. By employing metallic zinc as the anode, specific capacities of 820 mAh/g and 5854 mAh/cm^3^ can be achieved, though it is important to note that these capacities refer only to the anode and not to the full battery [4].

The capacity of zinc metal batteries is limited by major side reactions, particularly hydrogen evolution and uneven zinc deposition, which lead to the formation of dendrites on the anode [4]. These tree‐like metallic structures can penetrate the separator and cause short‐circuiting, resulting in battery failure [4]. Dendrite growth arises from zinc ion depletion near the electrode surface and increases the electrode surface area, thereby accelerating unwanted side reactions and causing the loss of active material [4]. Mitigating dendrite formation is crucial for enhancing the safety, stability, and overall performance of zinc‐based batteries, thus leading to their viability as a commercial battery system [4].

One of the most promising strategies, to mitigate both dendrite formation and gas evolution in ZIBs, is the introduction of additives into the electrolyte [4]. Chelating agents like ethylenediaminetetraacetic acid (EDTA) [7], sodium tartrate [8], or sodium citrate, along with solvents such as methanol [9] and glycerol [10], help reduce the amount of active water. Specific additives, including indium(III) sulfate and boric acid, promote crystal growth along the 002 crystal plane of the electrode, acting as artificial crystallization nuclei [11, 13]. Organic solvents like DMSO [14], diethyl ether (Et_2_O) [15], and acetonitrile [16] can adsorb onto dendrite tips, blocking further zinc deposition [11]. Additionally, polymers such as polyethylene glycol (PEG) [17] or polyethylene oxide (PEO) [18] form a film, which acts as a barrier on the electrode surface, minimizing water molecule interaction and controlling passivation [11]. These strategies effectively minimize dendrite growth, enhancing both the performance and safety of zinc metal batteries [11]. Despite the various additives developed and tested to mitigate dendrite formation, satisfactory reduction of dendrite formation and hydrogen evolution has not been achieved [11]. Because of this, in many recent studies, researchers have pursued a single all‐in‐one additive that can simultaneously suppress dendritic growth, coordinate Zn^2+^ ions, and stabilize the electrode interface. Although this idea is attractive, such multifunctional molecules often require elaborate syntheses, raise material costs, and may introduce environmental and toxicological concerns [19, 27].

Recent research highlights the potential of EDTA and SDBS as electrolyte additives to improve the performance and stability of zinc‐ion batteries in mildly acidic aqueous electrolytes. EDTA acts as a chelating agent, modifying the environment of hydrated Zn^2+^ ions to suppress side reactions and promote uniform zinc deposition by providing nucleation sites on the zinc foil surface [28]. This chelation prevents the formation of insulating Zn_4_(OH)_6_SO_4_•H_2_O (ZHS) by‐products, enhancing coulombic efficiency to 99.2% at high current densities [29]. In acidic solutions, EDTA‐2Na creates protective layers on zinc anodes, allowing stable cycling for over 1700 h at 1 mA/cm^2^ [30]. SDBS, an anionic surfactant, suppresses dendrite growth through interfacial adsorption, achieving dendrite‐free zinc deposition with 85% capacity retention after 1000 cycles at 1 A/g in Zn//MnO_2_ cells [31]. High concentrations of SDBS improve the morphology of deposited zinc and interfacial stability, enhancing battery performance [32]. Systematic screening confirms SDBS’s effectiveness and its mechanism’s applicability to other anionic surfactants [33]. Reviews indicate that additives like EDTA and SDBS are cost‐effective and safe options to regulate zinc deposition and minimize side reactions in mild acidic electrolytes, offering a promising pathway for practical applications [19].

We took a different approach in a recent publication [34]. We believe a more sustainable and cost‐effective approach is to combine several specialized additives, each tuned to perform a specific function at the interface, while relying on readily available and inexpensive compounds. We tested an additive mixture that combines several beneficial properties into a single electrolyte, unlike previously published mixtures [34]. Previous additive mixtures, such as those presented by Chen et al., emphasize the symbiotic effects of D‐mannose and sodium lignosulfonate [35]. These additives alternate in their adsorption at the zinc surface, creating a protective layer [35]. Min et al. introduced polyethylene oxide [36], while Wang et al. reported on a mixture of dimethylacetamide and trimethyl phosphate [37]. Both of these additives work to reduce the activity of free water and mitigate water‐induced side reactions [37]. Additionally, Motlagh et al. proposed a blend of the ionic liquid 1‐butyl‐1‐methylpyrrolidin‐ium dicyanamide and ethylene glycol, targeting an increase in the electrochemical stability window and a decrease in water‐induced side reactions [38].

We combined in our previous publication [34] SDBS, an additive that adsorbs onto the zinc surface, with EDTA, which we postulated modifies the solvation of Zn^2+^ ions. We used an SDBS concentration of 5.5 mM. SDBS adsorbs onto the zinc electrode, forming a protective layer that promotes uniform crystallization. Additionally, we employed 4.5 mM EDTA to inhibit water splitting. EDTA reduces the activity of water at the zinc surface. The combination of both additives exhibits a synergistic effect, resulting in improved performance compared to cells with either additive used alone. We proposed that the handover of zinc ions between EDTA in solution and the SDBS layer contributes to this enhancement. Our tests on the Zn//Zn symmetric cell were conducted for 3850 h and involved 1925 cycles at a current density of 1 mA/cm^2^ and a capacity of 1 mAh/cm^2^. Ultimately, we also tested zinc‐manganese dioxide (Zn‐MnO_2_) full cells, which demonstrated a capacity retention of 52% over 400 cycles [34].

Work establishing and optimizing an additive blend demands, in practice, a deep mechanistic understanding of how surfactants, chelators, and other modifiers interact at the electrode electrolyte interface. By unraveling these synergistic effects at the molecular level, we can rationally design additive blends that achieve uniform zinc plating without compromising on sustainability or economic viability [19, 20]. In this publication, we delve into the mechanism by which EDTA and SDBS function synergistically as a mixture. We explore how these two compounds interact at a molecular level, potentially leading to enhanced efficacy in various applications. Furthermore, we present compelling evidence that substantiates the proposed mechanism outlined in our previous publication, offering a more comprehensive understanding of their combined effects. Through detailed experimental findings and analysis, we aim to clarify the roles each component plays in this mixture, ultimately contributing to the broader field of chemical interactions.

## 2 Results and Discussion

### 2.1 Understanding the Additive Blend

In aqueous ZnSO_4_ electrolytes, the cycling stability of Zn metal electrodes is limited by inhomogeneous deposition and parasitic side reactions, most notably hydrogen evolution. In the absence of additives, symmetric Zn//Zn cells typically fail within several hundred hours under moderate current densities, reflecting continuous surface roughening and loss of active area (Figure S1). The introduction of single‐component additives provides only partial mitigation of these effects. For instance, the addition of sodium dodecylbenzenesulfonate (SDBS) extends the cycling lifetime to approximately 1500 h, while ethylenediaminetetraacetic acid (EDTA) yields no improvement with around 42 h under identical conditions. These observations indicate that both components influence interfacial processes, albeit to different extents.

When combined, however, SDBS and EDTA give rise to a markedly different behavior. Symmetric Zn//Zn cells containing the additive blend sustain stable cycling for up to 3850 h (1925 cycles) at 1 mA/cm^2^ and 1 mAh/cm^2^ (Figure S1). The initial overpotential of around 110 mV decreases to around 90 mV within the first few cycles and remains comparatively stable over extended cycling. Only after approximately 3500 h does a gradual increase in overpotential become apparent, which may be associated with gas accumulation or progressive changes in the electrode surface. The substantial extension in lifetime compared to the individual additives suggests a nonadditive, synergistic interaction between SDBS and EDTA.

This behavior is consistent with a modification of Zn deposition dynamics. The improved cycling stability and relatively stable overpotential point to a more uniform plating/stripping process, which in turn may suppress the formation of high‐surface‐area structures and reduce the rate of hydrogen evolution. While our previous work [34] introduced this additive system and demonstrated its beneficial effect in both symmetric and full cells, the origin of the apparent synergy remains insufficiently understood. The results presented here aim to clarify the underlying mechanisms that govern this combined effect and to provide a more detailed picture of how interfacial processes are altered in the presence of both components.

**FIGURE 1:**
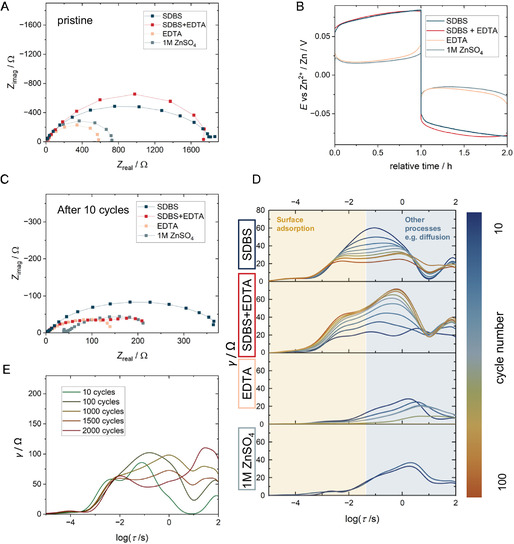
(A) Nyquist plot of the impedance spectra which were measured before the cycling experiment of cycling test of symmetrical Zn//Zn cells with an additive blend consisting of SDBS and EDTA at 1 mA/cm^2^ and 1 mAh/cm^2^ shown in Figure S1. (B) Enlarged view of one cycle for each electrolyte composition from the cycling experiment in (A). (C) Nyquist plot of the impedance spectra which were measured after the 10^th^ cycle of the cycling experiment in (Figure S1). (D) DRT plots of the impedance spectra which were measured after every 10^th^ cycle of the cycling experiment in (Figure S1). (E) DRT plots of the impedance spectra after certain cycles of the cycling experiment in (A) for the measurement with SDBS and EDTA as additives.

The symmetrical Zn//Zn cells using only 1M ZnSO_4_ as the electrolyte exhibit an overpotential of approximately 30 mV, as shown in Figure 1B. In contrast, the cells with added SDBS show an overpotential of around 80 mV, while the cells containing a mixture of SDBS and EDTA have an overpotential of about 100 mV. Thus, the addition of SDBS to the electrolyte of the cells leads to a 2.5‐fold increase in overpotential. This increase can be attributed to the adsorption of SDBS molecules and thus formation of a steric barrier on the electrode surface, which hinders the transport of reactants, i.e., affects the kinetics of the charge transfer reaction. As a result, the plating mechanism becomes the rate‐determining step instead of diffusion [36, 39]. This shift allows for a more homogeneous plating process, as the plating reaction now depends on the reaction itself rather than the concentration gradient from the electrode to the bulk solution. In a diffusion‐controlled environment, the shorter distance to a dendrite compared to the rest of the electrode leads to more heterogeneous plating and an increase in dendrite formation. However, when the plating reaction is the limiting factor, the concentration of zinc ions remains sufficient around the zinc electrode, ensuring uniform plating. This ensures a more homogeneous plating process overall. Such insights show the role of surfactants in altering electrode dynamics and underscore the importance of understanding their interactions within electrochemical systems. The absence of a distinct nucleation overpotential and the rise of a dominant growth overpotential in SDBS‐containing electrolytes show that Zn deposition shifts from a nucleation‐controlled regime to a growth‐controlled regime on the uniformly surfactant‐covered surface [40]. While macroelectrode measurements cannot entirely exclude mass‐transport contributions to absolute kinetic parameters, the absence of current plateaus in the Tafel plots and the nonmonotonic, composition‐dependent trends in charge–transfer resistance confirm that interfacial kinetics (rather than bulk diffusion) constitute the dominant rate‐limiting factor in the presence of SDBS and EDTA. Future studies employing ultramicroelectrodes under hemispherical steady‐state diffusion conditions or rotating disk electrodes with Koutecký–Levich analysis would enable rigorous separation of charge–transfer and mass‐transport contributions, further validating the mechanistic framework proposed here. In situ electrochemical quartz crystal microbalance (QCM) measurements would additionally provide direct, potential‐resolved quantification of additive adsorption coverage, establishing a quantitative link between surface occupation and the kinetic signatures identified by EIS and Tafel analysis.

We observe a similar trend to the one seen with the overpotential in our EIS measurements, shown in Figure 1A,D. The measured symmetric Zn//Zn cells containing SDBS, with and without EDTA, exhibit a notably high impedance in EIS, especially after ten cycles of cycling. While EDTA‐only and SDBS‐only adlayers reorganize into more permeable films that progressively lower charge transfer resistance (*R*_ct_), the SDBS + EDTA dual‐binding motif drives continuous densification of the mixed interphase, thereby increasing the DRT‐derived adsorption‐region resistance [41]. After ~10 cycles, the marked drop in *R*_ct_ for the SDBS + EDTA system indicates that EDTA intercalates into the initial SDBS film and drives its reorganization into a thinner, ion‐permeable adlayer that facilitates Zn^2+^ charge transfer.

Furthermore, all of the semicircles in the Nyquist plot of the EIS are depressed, indicating a nonideal behavior in the system, but also many overlapping or simultaneously occurring reactions. The increased impedance can be attributed to the formation of a distinct adsorption layer on the electrode surface. Fitting a model to our data is not feasible due to the depression of the semicircles, as illustrated in Figure 1A,C. Fitting equivalent circuits to strongly depressed semicircles in EIS data is often unreliable because such spectral features deviate significantly from idealized semicircular responses, indicating distributed or fractal relaxation processes that cannot be accurately represented [42, 43]. Consequently, the use of conventional equivalent circuit models in these cases can lead to overparameterization and poor physical interpretability, as also emphasized by Vadhva et al. [44]. Because the strongly depressed semicircles reflect fractal‐like, distributed interfacial relaxations that defy unique mapping onto ideal R–C(R) or R–CPE elements [45, 46], we have avoided conventional equivalent‐circuit fitting and instead extracted mechanistic insights through high‐resolution DRT analysis. The deviation from ideal semicircular behavior arises from electrode surface roughness, adsorption heterogeneity, and frequency dispersion (broadening of DRT peaks reflecting nonideal RC behavior [47]), all processes that are present in our system [48]. This layer significantly obstructs both charge transfer processes and the diffusion of zinc ions through the adsorbed film, making the zinc plating reaction the rate‐limiting step.

To differentiate the many overlapping contributions in the Nyquist plots, we performed DRT analysis to distinguish between the different reactions occurring within the system. The resulting curves are presented in Figure 1D, which displays the cells without additives, those with individual additives, and the mixtures of the additives. In the DRT plots, the cells whose electrolyte includes SDBS, a peak is visible at shorter relaxation times of 10^−2^ s. In this time range, peaks occur for adsorption processes, such as a needed SDBS adsorption on the zinc surfaces [49, 50]. In corrosion studies, the impedance response of SDBS‐containing systems demonstrates strong frequency dependence, particularly in the low‐frequency regime, where impedance values can reach exceptionally high levels. This behavior is attributed to the formation of organized molecular structures that impede charge transfer processes [49, 50]. The complex impedance responses indicate that the SDBS layer plays a crucial role in determining the overall impedance mechanism, with the surfactant creating barriers to ionic conduction through its organized interfacial structure.

All DRT curves exhibit peaks in the relaxation time region around 1 s. This peak is associated with diffusion processes and is more pronounced in the cells containing the additive mixture compared to the other cells. All cells, except those containing the additive mixture, exhibit decreasing DRT peaks throughout the depicted cycle time. This decline in peak intensity can be attributed to the undesired dendrite formation, which increases the effective surface area. For the cells with EDTA and those without additives fewer spectra could be obtained, as they experienced short circuits before completing 100 cycles. As a result, the resistance associated with the processes decreases. In contrast, the peak intensity of the cells containing the additive mixture increase during the first 100 cycles. We attribute this increase in peak intensity to reduced active surface area caused by more heterogeneous zinc plating and fewer dendrites, coupled with some blocking caused by H_2_ bubbles and ZHS adsorption. This result indicates a more homogeneous and desirable zinc plating reaction, while also highlighting the presence of other side reactions. This behavior reflects the behavior of the overpotentials observed in the cycling experiments. The DRT response regions are assigned based on the literature. The window of *τ* ≈ 10–100 ms corresponds to the adsorption of organic additives, while slower processes reflect diffusion and solid electrolyte interphase (SEI) formation [51, 52]. The combined EDTA/SDBS system demonstrates a synergistic effect with peak evolution that is cycle‐dependent, rather than exhibiting simple competitive behavior. This confirms the presence of cooperative interfacial modulation. Additional experiments, such as QCM measurements or concentration variations, are considered outside the scope of this manuscript, as the DRT assignments are already well‐supported by existing literature, including dynamic QCM adsorption studies [53].

The EIS and DRT results support the hypothesis from the cycling results, suggesting that SDBS creates an adsorption layer on the surface of the zinc electrode. This layer makes the plating reaction, rather than diffusion, the rate‐limiting step, leading to more uniform plating. Post‐mortem light‐microscopy of Zn electrodes after cell failure (Figure S14) shows dendritic deposits in all cases, with EDTA‐only yielding localized coarse protrusions and SDBS + EDTA producing a more compact, uniformly granular morphology, thereby confirming that additive composition critically tunes Zn growth patterns.

**FIGURE 2:**
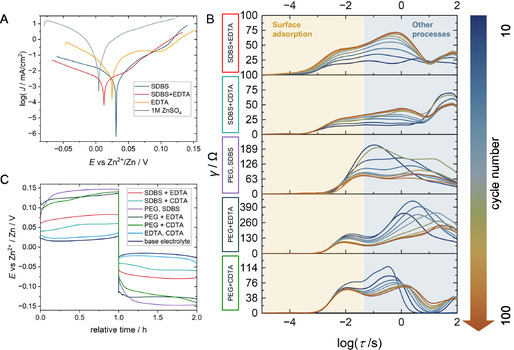
(A) Tafel plots derived from LSV measurements with different electrolytes measured in symmetrical Zn//Zn cells with Zn wire pseudo reference electrodes. (B) DRT plots of the impedance spectra which were measured after every 10^th^ cycle of the cycling experiment for different additive formulations from cycling experiments of symmetrical Zn//Zn cells at 1 mA/cm^2^ and 1 mAh/cm^2^ from Figures S1–S8. (C) Enlarged view of one representative cycle for each electrolyte composition from the cycling experiment of symmetrical Zn//Zn cells with different additive blends containing surface active additives at 1 mA/cm^2^ and 1 mAh/cm^2^ from Figures S1–S8.

To further validate that SDBS is adsorbing on the electrode surface from a different perspective, we conducted linear sweep voltammetry (LSV) measurements to determine the corrosion potential and corrosion current and then calculated the surface coverage as described in the supporting information equation 1 [54]. The Tafel plots of these measurements are depicted in Figure 2A for the base electrolyte, the individual additives, and the mixture of SBDS and EDTA. The measurements indicate that the addition of additives reduces the corrosion current by two orders of magnitude and increases the corrosion potential by at least 10 mV, which, in turn, decreases corrosion in the system. Notably, the measurements that included SDBS resulted in the lowest corrosion currents and the highest coverage of approximately 99% for both the mixture and the SDBS alone. This suggests that SDBS forms a protective layer on the electrode surface, providing further validation for our proposed mechanism. The EDTA electrolyte exhibited approximately 96% surface coverage, suggesting that EDTA also tends to adsorb at the surface to some extent. It is important to clarify that the high surface coverage mentioned does not create a static, impermeable layer. Instead, the additives form a dynamic adlayer that rearranges reversibly in response to potential changes during plating and stripping. This rearrangement creates temporary ion channels that allow uniform access to Zn^2+^ ions while selectively passivating high‐energy defect sites.

EDTA and SDBS play important roles not only at the interface but also in the bulk electrolyte. They reduce the desolvation energy of Zn^2+^ ions by forming EDTA–Zn^2+^ complexes and reorganizing the hydrogen bonding network. These effects work in conjunction with the DRT‐resolved adsorption dynamics at the electrode surface, leading to notable improvements in plating morphology and cycling stability.

### 2.2 The Influence of Surfactants

The results and findings from the corrosion experiments, combined with the EIS and cycling data, suggest that a certain level of surface coverage is necessary to inhibit the plating reaction. This reaction becomes the rate‐determining step, which leads to homogeneous plating and extended cycling performance. To validate this hypothesis, we introduced another surface‐active additive with a different chemical composition, polyethylene glycol 300 (PEG300). This addition demonstrates that the cycling performance is influenced not by chemical effects but rather by the kinetic effects discussed earlier. PEG300 is recognized for its properties as a surface‐active additive in zinc‐ion batteries, known for enhancing performance and stability [17]. Our observations indicated that, similar to the results obtained with SDBS, the presence of the PEG300 additive led to an increased overpotential compared to the baseline measurements without any additives, as shown in Figure 2C. Additionally, we noted a significant rise in the overall impedance of the system, particularly in the initial stages of the testing. This elevated impedance at around 10^−2^ s suggests that this additives influence the charge transfer dynamics within the battery. Moreover, we recorded an increased number of cycles that the cells could withstand before experiencing a short circuit.

Looking at the data (see Table 1), one can see that SDBS combined with the mixture of SBDS and EDTA resulted in a total of 1950 cycles, while SDBS and PEG300 reached 305 cycles. The combination of PEG300 with EDTA produced the highest performance with 2603 cycles (Figure S2). Because of this and the increase from only 43 cycles obtained when using only 1 M ZnSO_4_ without additives, we conclude that in cells with PEG300, the zinc plating reaction at the electrode becomes the rate‐limiting step, which results in less dendrite formation due to the reduced influence of diffusion on the reaction kinetics. The observed voltage fluctuations during symmetric‐cell cycling arise from transient adlayer rupture and repair, localized dendrite contact, and bubble detachment, all of which are markedly dampened by the self‐healing additive interphase, highlighting its critical role in interfacial stabilization.

**TABLE 1:** Summary of key parameters used throughout the manuscript for all used additive combinations and without additives.

Surface additive	Chelate additive	Max. cycle number	Overpotential, mV	Contact angle	Coverage
—	—	43 ± 6	30	112.1°	0%
SDBS	—	773 ± 37	80	16.9°	99%
SDBS, PEG300	—	305 ± 60	150	28.0°	99%
SDBS	EDTA	1925 ± 96	85	16.9°	99%
SDBS	CDTA	550 ± 68	60	36.9°	99%
SDBS	His	541 ± 46	50	59.3°	99%
SDBS	Zn(OAc)_2_	1011 ± 148	85	22.3°	99%
PEG300	EDTA	2600 ± 337	125	26.0°	99%
PEG300	CDTA	710 ± 33	125	39.3°	99%
—	EDTA	42 ± 7	30	59.1°	96%

To establish that PEG300 adsorbs onto the electrode, we conducted EIS measurements and calculated the DRT curves, allowing for a comparison between PEG300 and SDBS. Both PEG300 and SDBS displayed comparable characteristics in their spectroscopic profiles (see Figure 2), in particular a pronounced peak at low relaxation times (*τ*). This specific peak at around 1 s is a critical indicator of adsorption processes occurring at the electrode interfaces, suggesting a strong interaction between the additives and the surface [49, 50]. Further examination revealed that this low‐*τ* peak was notably absent in systems that contained solely chelating agents or in electrolytes devoid of any additives. This absence indicates that the adsorption phenomenon is closely tied to the chemical properties of PEG300 and SDBS on the electrode. Thus, the findings emphasize the significant role of PEG300 and SDBS in modifying electrode behavior through adsorption, providing a clearer understanding of their functional mechanisms in complex electrochemical systems. Furthermore, corrosion measurements, i.e., linear sweep voltammograms (LSV), were conducted. From them, Tafel plots were made (S12 and S13), and the surface coverages were calculated for SDBS, EDTA, and their mixtures. All mixtures containing surface‐active additives (SDBS and/or PEG300) demonstrate coverages of at least 99%. However, no correlation could be established between coverage and the possible cycle number in the additive blends containing these surface additives. This further validates our proposed mechanism, which suggests that once the electrode reaction becomes the rate‐determining step, any additional coverage does not have an effect.

Building on the previous results, we now show that this saturation effect is further supported by our measurement series, where we introduced PEGs with longer chain lengths, as shown in the supporting information in Figure S10. We used PEG300, PEG600, and PEG1000 as surfactant‐type additives, and for all of them, we observed similar DRT peaks and overpotentials, but with increasing coverages corresponding to the increasing chain length. However, the achieved cycle number decreased as shown in Table 1, highlighting that there is a combination of factors that allows for optimal cycling.

### 2.3 The Influence of EDTA and Chelating Agents

**FIGURE 3:**
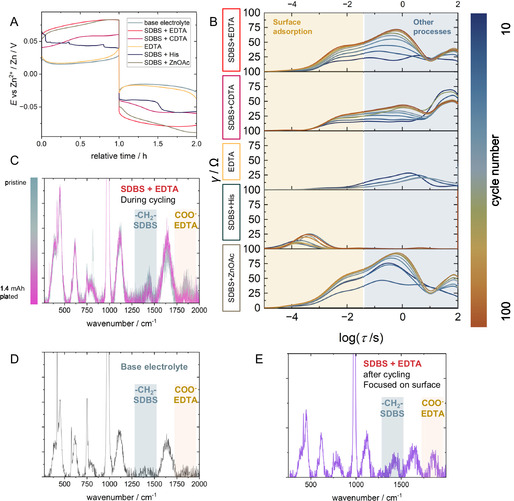
(A) Analysis of a single cycle for various electrolyte compositions from the cycling experiment of symmetrical Zn//Zn cells with different additive blends, which include chelating agents, at 1 mA/cm^2^ and 1 mAh/cm^2^ is presented in Figures S1–S8. (B) DRT plots of the impedance spectra which were measured after every 10^th^ cycle of the cycling experiment for different additive formulations from the cycling experiment of symmetrical Zn//Zn cells with different additive blends containing chelating agents at 1 mA/cm^2^ and 1 mAh/cm^2^ from Figures S1–S8. (C) operando Raman spectra taken during plating at 0.7 mAh/cm^2^ until Zn corresponding to 1.4 mAh has been plated (peaks assigned in Figure S11). (D) In situ Raman base electrolyte (peaks assigned in Figure S11). (E) In situ Raman mixture after cycling (peaks assigned in Figure S11).

In addition to the surface hindering mechanism provided by the surfactant, we also need to understand the role of the chelating agent/additive EDTA in our blend. EDTA not only participates in the electrode reaction but also increases the potential cycle number compared to measurements taken with just a surface‐active additive as shown by us in our previous publication [34]. To investigate the influence of EDTA, we replaced it with different chelating agents with varied chemical composition and zinc complexation constants *K*. We replaced EDTA with either trans‐1,2‐diaminocyclohexane‐N,N,N′,N′‐tetraacetic acid (CDTA), L‐histidine (His), and zinc acetate (Zn(OAc)_2_). Zn(OAc)_2_ has the lowest *K*‐value at 1.5, which is the same as that of the conducting salt ZnSO_4_ in the electrolyte [55, 56]. In comparison, the complexation constant of His is higher at 11.8, while the complexation constants for EDTA and CDTA are both 16.5 [57, 59]. The different chelating agents were combined with SDBS in an electrolyte alongside SDBS to investigate their effects. The cycling experiments (Figure 3A) of these cells demonstrate that the highest and similar overpotentials are around 85 mV for the cells containing SDBS + EDTA and SDBS + Zn(OAc)_2_. The voltage profiles for the combinations of SDBS + EDTA and SDBS + Zn(OAc)_2_ also appear similar. In contrast, the cells with SDBS + CDTA exhibit lower overpotentials of 60 mV. Furthermore, the cells with SDBS + His show the lowest overpotentials at 50 mV, along with a distinct voltage profile.

We also conducted EIS measurements and ran DRT analysis. The mixtures of SDBS + EDTA and SDBS + Zn(OAc)_2_ appear similar, exhibiting the same peaks and comparable peak heights. In contrast, SDBS + CDTA shows a similar adsorption peak around 10^−2^ s; however, the peak at 1 s is smaller, while the peak at 100 s is higher. All these mixtures demonstrate an increase in peak heights over the first 100 cycles. The DRT curves for the cells with SDBS + His look significantly different, featuring only one notable peak at 10^−4^ s. with a maximum height of 25 Ω. The coverages calculated from the Tafel plots depicted in Figure S12 for all additive mixtures range between 96% and 99%.

Among the tested electrolyte additive combinations, the cycling stability depended significantly on the additive pair as shown in Figures S1–S8. The system containing SDBS + CDTA completed 550 cycles before failure, while the pairing of PEG300 and CDTA extended the lifespan to 711 cycles. A further improvement was observed for the SDBS and Zn(OAc)_2_ system, which sustained 1011 cycles, representing the most durable performance among all formulations.

Overall, we conclude from our measurements that the performance of the chelating additives is not directly proportional to the zinc ion complex constant *K* shown in Table S2 nor is it indicative of their ability to chelate zinc ions. In fact, we observe a stronger relationship between cycling performance and the availability of sterically unhindered acetate groups, as seen in EDTA or Zn(OAc)_2_. EDTA and Zn(OAc)_2_ were used at the same molar concentration to ensure stoichiometric consistency between the chelator and the metal precursor. Although EDTA contains a higher nominal content of acetate equivalents, the resulting variation in OAc^−^ concentration was not adjusted, as acetate was not anticipated to significantly affect the investigated process under the applied conditions.

To further investigate the role of the acetate group and provide additional evidence for the presence of SDBS at the surface, we conducted operando Raman measurements during plating at 0.7 mAh/cm^2^. Areal capacity in the operando cell was limited to 0.7 mAh/cm^2^ to ensure reliable signal quality and avoid excessive polarization arising from the modified cell geometry, while staying in a regime where electrode utilization, potential profiles, and reaction mechanisms are comparable to those obtained at 1 mAh/cm^2^ in the coin cells, so that the trends can be directly correlated [60, 61]. In the Raman spectra depicted in Figure 3, we observe an alkyl CH_2_ deformation peak at a wavenumber of around 1440 cm^−1^ (highlighted in gray) [33, 62], as well as a peak indicative the interaction of a COO^−^ group with a zinc surface, as previously observed by Hadjiivanov et al. (highlighted in orange) [63]. Neither peak appears to be in measurements without SDBS or EDTA, as illustrated in Figure 3D. We have assigned the CH_2_ peak to the alkyl chain of SDBS and the COO^−^ peak to EDTA, since these are the only compounds in the system that contain these groups. During the plating process, the peaks decrease in intensity, as observed in the operando measurement. We attribute this phenomenon to the changing location of the zinc surface as more zinc gets plated and the zinc surface expands. To confirm this, we refocused on the zinc surface after the plating and measured another Raman spectrum, which is depicted in Figure 3E. Here, the CH_2_ and COO^−^ peaks show the same intensity as at the beginning of the cycling. The necessity to refocus the Raman laser is likely due to a spatial displacement of the adsorbate, which is consistent with the migration of zinc beneath the SDBS layer. This observation suggests that the SDBS layer does not serve as an impermeable barrier; rather, only a partial restraint to the plating process. We hypothesize that the acetate groups participate in the reactions at the electrode. We have already found initial evidence supporting this hypothesis in the increased surface coverage observed with the introduction of EDTA into the system, compared to the base electrolyte, as well as between the electrolytes containing only SDBS and those that include both SDBS and EDTA. This supports our proposed mechanism, indicating that both the surfactant SBDS and the COO^–^ group are present at the surface throughout the entire plating process and actively participate in the plating mechanism. These two additives likely interact with each other, resulting in a synergistic effect that contributes to the previously observed cycle numbers [34].

Unlike traditional overpotential or spectroscopic analyses that correlate the presence of additives with performance but fail to clarify overlapping interfacial phenomena, the combined DRT–Raman approach utilized in this study quantifies the distinct kinetic signatures of EDTA and SDBS coadsorption. This method uncovers their dynamic interplay and mutual influence at sub‐second timescales, a mechanistic insight that neither technique could achieve independently.

To get the large observed increase in cycle life, there is an interplay between the chelating agent and the surfactant, or perhaps a competition of some sorts. Our hypothesis is that upon reducing the zinc ions to zinc metal, the now neutrals atoms will be deposited onto the zinc surface in a facile manner. This could be, for example, a stepped handover from EDTA to SDBS to the surface.

To establish that this proposed mechanism has plausibility, illustrative DFT calculations were done. This was done by constructing appropriate complexes in silico and calculating energy differences, as seen in Table 2, both in gas phase and in implicitly solvated water. An initial look shows that there is a very strong interaction between the chelating agents studied, EDTA and CDTA, and Zn^2+^. Similarly, there is also a strong interaction between SDBS and Zn^2+^. Though upon getting solvated, this interaction strength drops considerably. These facts are in line with what could be expected from electrostatics.

**TABLE 2:** Data from DFT calculations calculated energy differences of the depicted appropriate complexes in silico in the gas phase (Gas) and in aqueous media (Aq.).

		Gas		Aq.	
		Δ*E,* kJ/mol	Δ*G,* kJ/mol	Δ*E,* kJ/mol	Δ*G,* kJ/mol
Shortened SDBS	Na^+^ + SDBS^–^ → NaSDBS	−559.1	−521.4	−88.5	−54.0
	Zn^2+^ + SDBS^–^ → ZnSDBS^+^	−1681.6	−1641.8	−409.9	−370.3
	Zn^2+^ + 2 SDBS^−^ → Zn(SDBS)_2_	−2471.4	−2162.0	−1430.3	−1329.7
	Zn^0^ + SDBS^–^ → ZnSDBS^–^	−43.1	−10.8	−9.1	21.7
	Zn^0^ + 2 SDBS^–^ → Zn(SDBS)_2_^2+^	425.3	503.2	−71.0	29.6
EDTA	EDTA^4–^ + Zn^2+^ → ZnEDTA^2–^	−3869.8	−3806.8	−928.2	−873.8
	EDTA^4–^ + 2 Zn^2+^ → Zn_2_EDTA	−6276.7	−6179.3	−1327.5	−1236.2
	EDTA + Zn^0^ → ZnEDTA^4–^	−116.6	−80.4	−17.3	—a
CDTA	CDTA^4–^ + Zn^2+^ → ZnCDTA^2–^	−3611.5	−3554.9	−723.0	−677.9
	CDTA^4–^ + 2 Zn^2+^ → Zn_2_CDTA	−6475.4	−6354.4	−1652.3	−1550.0
	CDTA + Zn^0^ → ZnCDTA^4–^	−155.6	−117.9	−14.4	26.0

a There was a persistent imaginary mode in the implicit calculations.

Upon the reduction of Zn^2+^ to Zn^0^, there is a large change in the energetics of association. While there is still a small negative Δ*E* value, the Δ*G* values is slightly positive. Thus, in solution, one could expect that upon reduction the Zn would dissociate from the SDBS and EDTA molecules. However, as this is a small Δ*G* value, a small population could still exist. Thus, it is not unreasonable to hypothesize that the plating step can happen in a step wise manner, allowing for a smoother plating overall.

It would be interesting to run calculations of the full system to probe the system during zinc reduction and thus observe the plating through the surface adhered SDBS, though that is a very computationally expensive problem to tackle. In lieu of that, we can still reason about the plating mechanism. The Raman and EIS measurements have been able to localize the SDBS on the zinc metal surface. Due to the strong interactions of the DFT calculated species, we propose that they must act as a kinetic control.

## 3 Conclusion

In this work, we investigate the mechanism of our previously published additive blend for mildly acidic zinc‐ion batteries, which contains millimolar amounts of SDBS and EDTA as additives. We demonstrate insight into the complex chemical and electrochemical interactions that happen at the Zn electrode. We show through overpotential analysis, linear sweep voltammetry, DRT analysis, and Raman spectroscopy that both SDBS and EDTA adsorb onto the electrode surface and remain continuously present during the plating process. Additionally, we observed that the SDBS layer hinders the plating reactions, making the kinetics of these reactions the rate‐determining step, rather than the diffusion of Zn^2+^ from the bulk solution to the electrode surface. As a result, there is no reduction in the concentration of Zn^2+^ at the surface, allowing plating to occur uniformly across the entire surface rather than being localized at points closest to the bulk, such as dendrites. This leads to a more homogeneous plating process.

Moreover, we conducted tests using a different surface additive, PEG, which demonstrated that the distinct chemistry of the surface additive does not play a significant role in the process. Instead, it is essential that the electrode reaction is sufficiently hindered by a sufficient surface coverage to become the rate‐determining step. Additionally, we elucidated through an evaluation of various chelating agents that an important property for ensuring a long cycle life is not necessarily a strong chelating capability but rather the presence of sterically unhindered carboxyl groups (COO^−^) in the additive. We validated our findings by introducing various chelating additives, specifically trans‐1,2‐diaminocyclohexane‐N,N,N′,N′‐tetraacetic acid (CDTA), L‐histidine (His), and zinc acetate (Zn(OAc)_2_). Each of these additives contains different amounts of carboxylate (COO^−^) groups and exhibits distinct chelating strengths. This investigation not only provides valuable insights into the underlying mechanisms of our additive blend but also highlights a broader principle: to improve the cycle life of symmetric zinc–zinc (Zn//Zn) systems, it is essential to incorporate a surfactant that inhibits the electrode reaction alongside a source of COO^−^ groups.

In summary, the kinetic framework presented here offers a clear path for rational electrolyte design by isolating interfacial charge–transfer kinetics and near‐surface ion availability as the key parameters governing deposition stability. By assembling additive mixtures from simple, well‐characterized molecular motifs rather than relying on complex, highly fluorinated, or persistent chemistries, this approach decouples electrochemical performance from additive complexity and opens the door to low‐cost, chemically benign, and industrially compatible formulations. Looking ahead, the same mechanistic principles can be applied to other battery systems and integrated with systematic screening strategies to guide the discovery of new electrolyte blends. In doing so, this work not only establishes a practical kinetic basis for sustainable electrolyte engineering but also preserves the inherent advantages of aqueous zinc‐ion batteries, namely material abundance, affordability, environmental compatibility, and nontoxicity, while improving their performance through targeted additive design.

## Funding

This work was supported by the Bundesministerium für Wissenschaft und Forschung (03XP0481C).

## Conflicts of Interest

The authors declare no conflicts of interest.

## Supporting information

The authors have cited additional references within the Supporting Information [64, 78].

## Data Availability

The data that support the findings of this study are available from the corresponding author upon reasonable request.
