# Supplementary material for: Mechanistic Insights Into Surfactant‐Regulated and Carboxylate‐Mediated Uniform Zinc Plating
Source: ChemSusChem. 2026 Aug 3;19(15):e70945. doi: 10.1002/cssc.70945 (PMC13432792; doi:10.1002/cssc.70945)
Supplement: Supplementary file 1 — The authors have cited additional references within the Supporting Information [64, 78]. [file CSSC-19-e70945-s001.pdf]

Supporting Information

Mechanistic Insights into Surfactant-Regulated and Carboxylate-Mediated Uniform Zinc Plating

Joachim G.C. Hering, Erlendur Jónsson, Daniel Schröder

Methods

Electrolyte preparation

1M $ZnSO_4 \cdot 7\ H_2O$ and 0.2M $MnSO_4 \cdot 1\ H_2O$ were added to Millipore water and homogenized by ultrasound for five minutes. This was used as the reference electrolyte. Afterward, the additives were added, and the solution was homogenized again by ultrasound for five minutes. The pH value was then measured using a Seven Compact pH meter equipped with an InLab Routine Pro pH-Electrode, both from Mettler Toledo. All chemicals were bought from either Sigma-Aldrich or ACR in at least 99.9% purity. The concentration of the surface-active additive was at 5.5mM, while the concentration of the chelating agent was 4.5mM.

Cell preparation

Symmetrical cell measurements were conducted using CR2032-type stainless steel coin cells from MTI. The electrodes consisted of two zinc disks, each with a diameter of 15 mm and a height of 0.25 mm. These zinc foils (purity of 99.999%) were sourced from ChemPur and punched out for use. Before assembly, the disks were immersed in 1M hydrochloric acid for 10 s and then rinsed with Millipore water. A Whatman GF/A glass fiber separator with a diameter of 18 mm and a thickness of 125 µm was employed. Each cell contained 100 µL of electrolyte. The coin cells were sealed with an MSK-160E coin cell crimping press from MTI with the crimping conditions of 0.8 T. For the initial plating experiments, one of the zinc electrodes was replaced with a stainless steel spacer of 0.5 mm thickness. For each additive combination, three measurements were conducted.

Electrochemical tests

Electrochemical characterizations and electrochemical impedance spectroscopy (EIS) were performed using an electrochemical workstation from Arbin Instruments, equipped with an integrated Gamry 1010 Potentiostat for EIS measurements. The EIS measurements were conducted in a frequency range from 10 mHz to 1 MHz with an amplitude of 1 mV. Three points per decade were recorded. The measurements were conducted in a climate chamber at a temperature of 25°C. The linear sweep voltammetry measurements were performed with a SP150e Potentiostat from BioLogic at a scan rate of 1 mV/s. The distribution of relaxation time (DRT) data analysis used the software RelaxIS 3 by rhd instruments.

Operando Raman

Operando Raman spectra were collected during galvanostatic zinc plating in a custom rhd Instruments TSC Raman cell fitted with a sapphire-glass observation window. The working electrode was a zinc disk (15 mm diameter, 0.10 mm thickness, 99.9%) and the counter electrode a matching zinc foil. All components were filled with the mildly acidic zinc-ion electrolyte (1M $ZnSO_4$ with millimolar amounts of SDBS and EDTA). Electrochemical control was provided by a Biologic SP240 potentiostat. Galvanostatic plating was carried out at a current density corresponding to 0.7 mAh cm^{-2}, deposited over 2 h per cycle. During plating, operando Raman spectra were acquired every 10 min over the range 275 – 2000 cm^{-1} using a Renishaw inVia REFLEX confocal Raman microscope equipped with a 532 nm green excitation laser. The laser power at the sample was maintained below 1 mW to minimize heating. Each spectrum was collected with a 30 s integration time and one accumulation, yielding a spectral resolution of ~2 cm^{-1}. Raw spectra were processed by subtracting a polynomial baseline and applying a Savitzky–Golay smoothing filter. Peak positions and intensities were tracked in real time to monitor the evolution of interfacial species and surfactant adsorption throughout the zinc plating process. The non-operando measurements were conducted in the same setup but without cycling.

Computational

Density functional theory (DFT) calculations were performed at the B3LYP/def2-TZVP[1–6] level of theory with the D3BJ[7,8] dispersion correction. The molecules of interest were constructed as ions (in various protonation states) and ion-pairs, with Zn^{2+} and Na^{+}. Each pairing was optimized with a tight convergence criteria and verified to be a minimum via a frequency calculation. Due to an issue with the initial calculations, the finer grid option of defgrid3 was used throughout. In each case, the calculations were performed both in the gas phase as well as in an implicit solvent, here water as defined by the SMD[9] method. Orca version 6.1.1[10–15] was used throughout the calculations.

Contact angle measurements

Contact angle measurements were conducted using a Krüss DSA30 device. All measurements were performed on a freshly prepared zinc surface, similar to that used in the electrochemical experiments. Before taking the measurements, the disks were immersed in 1M hydrochloric acid for 10 seconds and then rinsed with Millipore water. For each measurement, 50 µL of the respective liquid was applied to the zinc surface. The contact angles were calculated using the DSA software provided by Krüss.

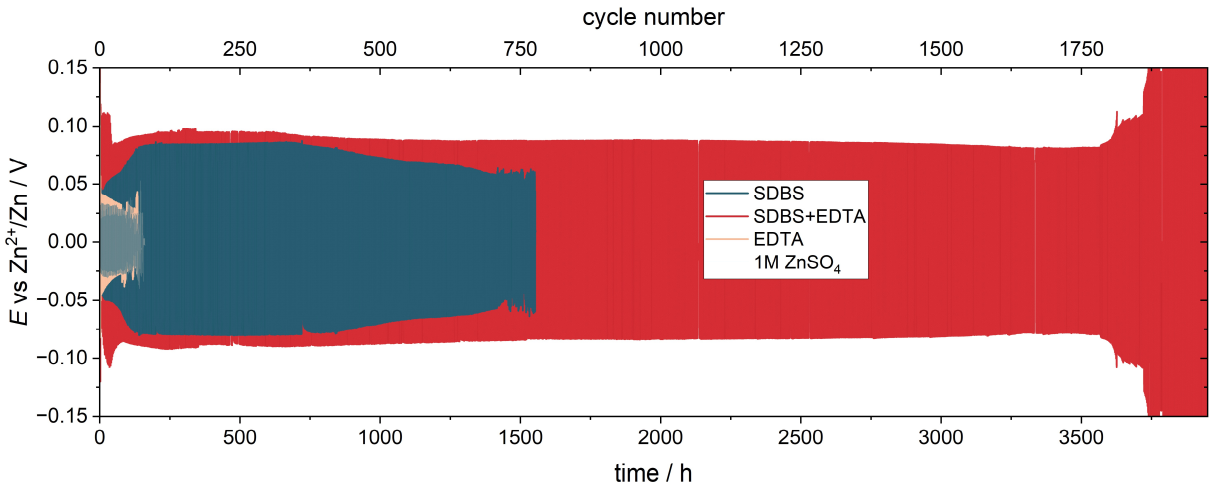

Figure S1: Results of cycling test of symmetrical Zn//Zn cells with an additive blend consisting of SDBS and EDTA at 1 mA/cm^2 and 1 mAh/cm^2. Previously published results (by the authors) are remeasured here, and the updated data are shown[34].

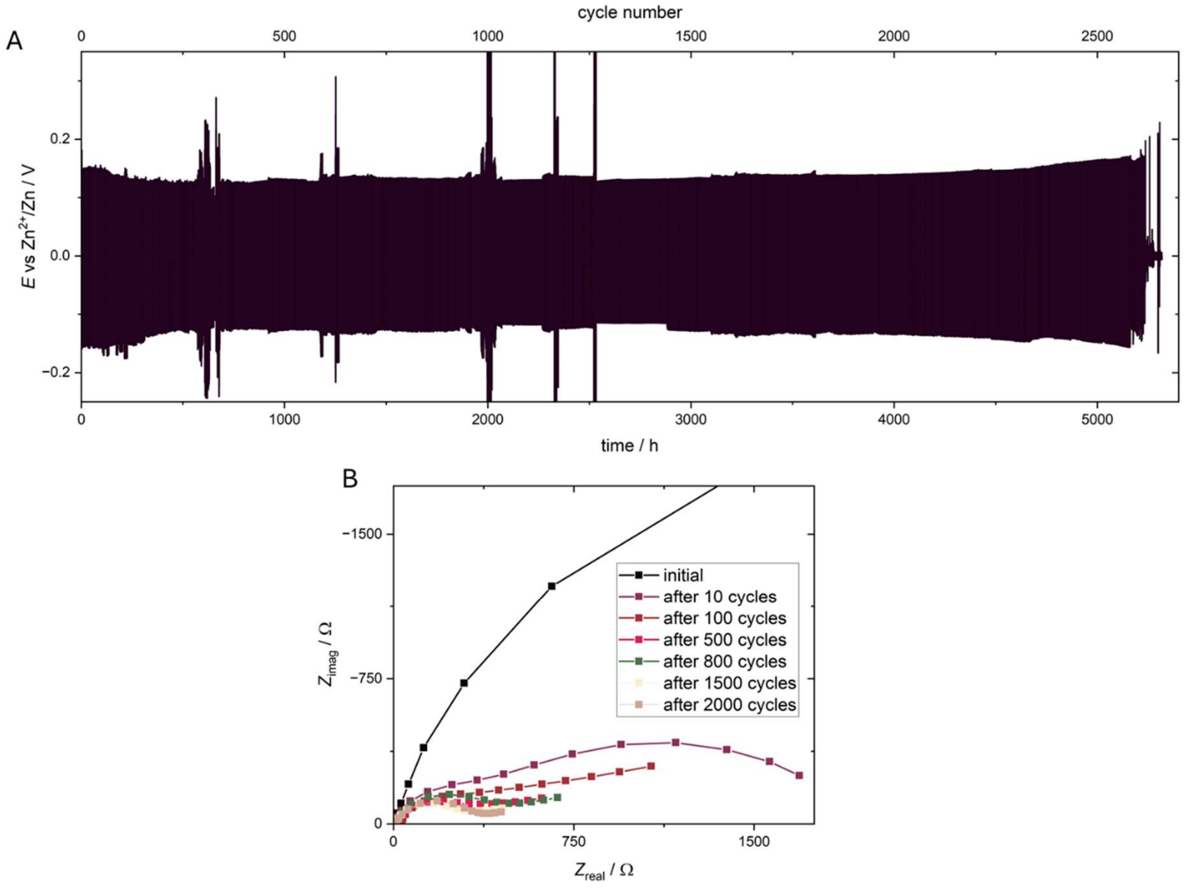

Figure S2: A) Cycling test of symmetrical Zn//Zn cells with an additive blend consisting of PEG300 and EDTA at 1 mA/cm^2 and 1 mAh/cm^2, B) Nyquist plot of the impedance spectra which were measured before/during the cycling experiment shown in (A).

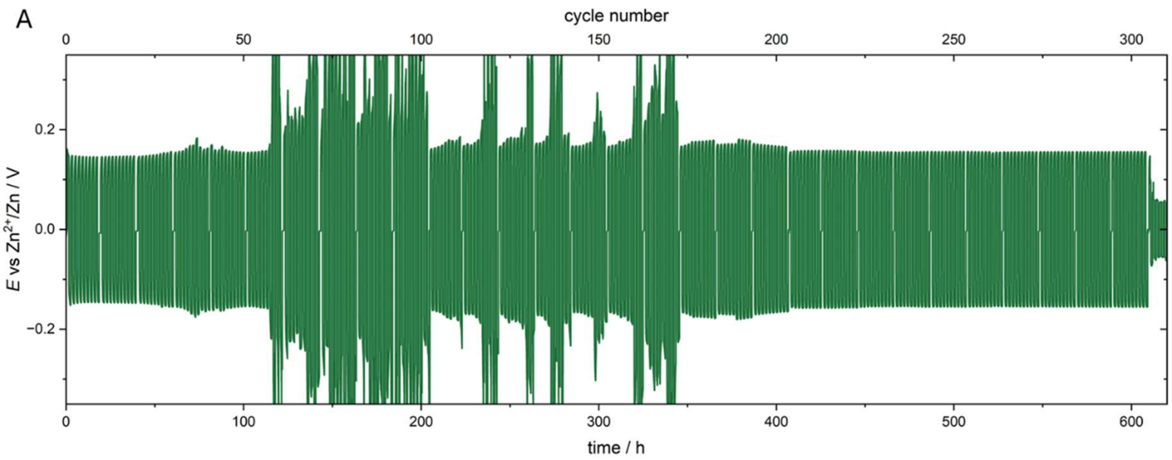

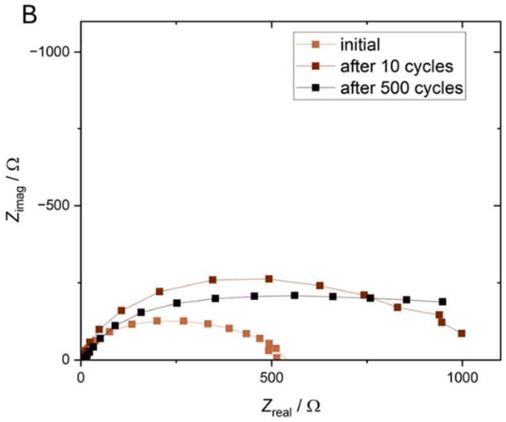

Figure S3: A) Cycling test of symmetrical Zn//Zn cells with an additive blend consisting of PEG300 and SDBS at 1 mA/cm^2 and 1 mAh/cm^2, B) Nyquist Plot of the impedance spectra which were measured before the cycling experiment for (A).

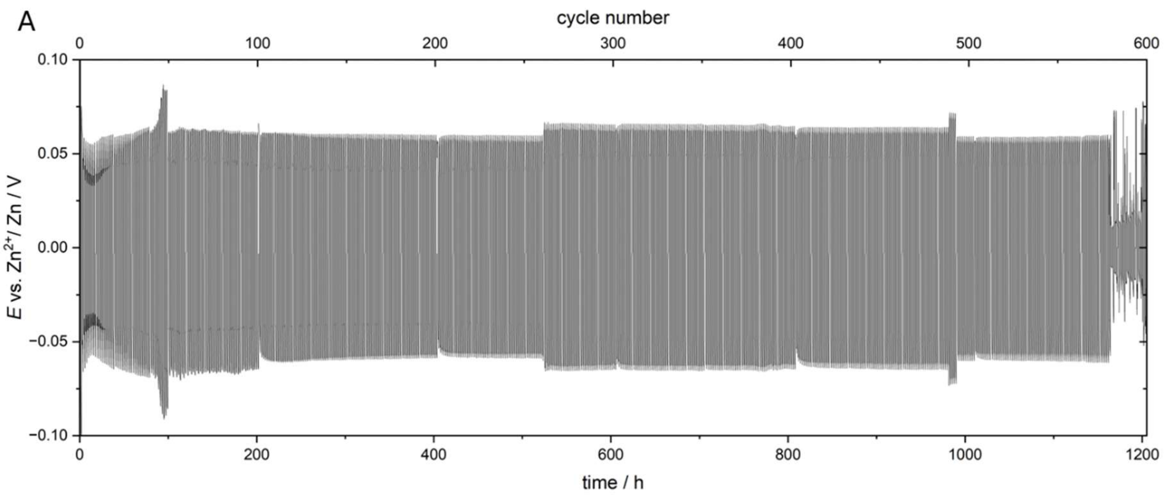

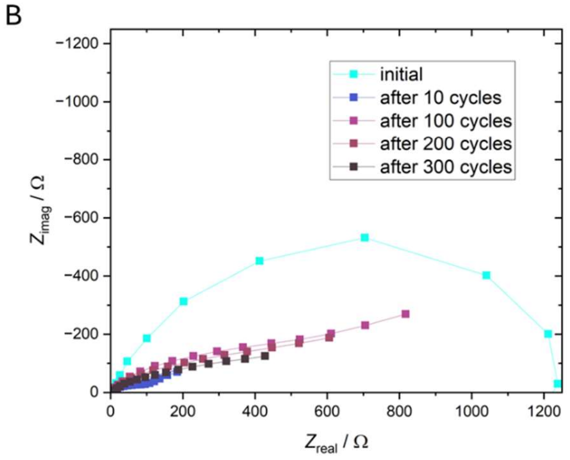

Figure S4: A) Cycling test of symmetrical Zn//Zn cells with an additive blend consisting of SDBS and CDTA at 1 mA/cm^2 and 1 mAh/cm^2, B) Nyquist Plot of the impedance spectra which were measured before the cycling experiment for (A).

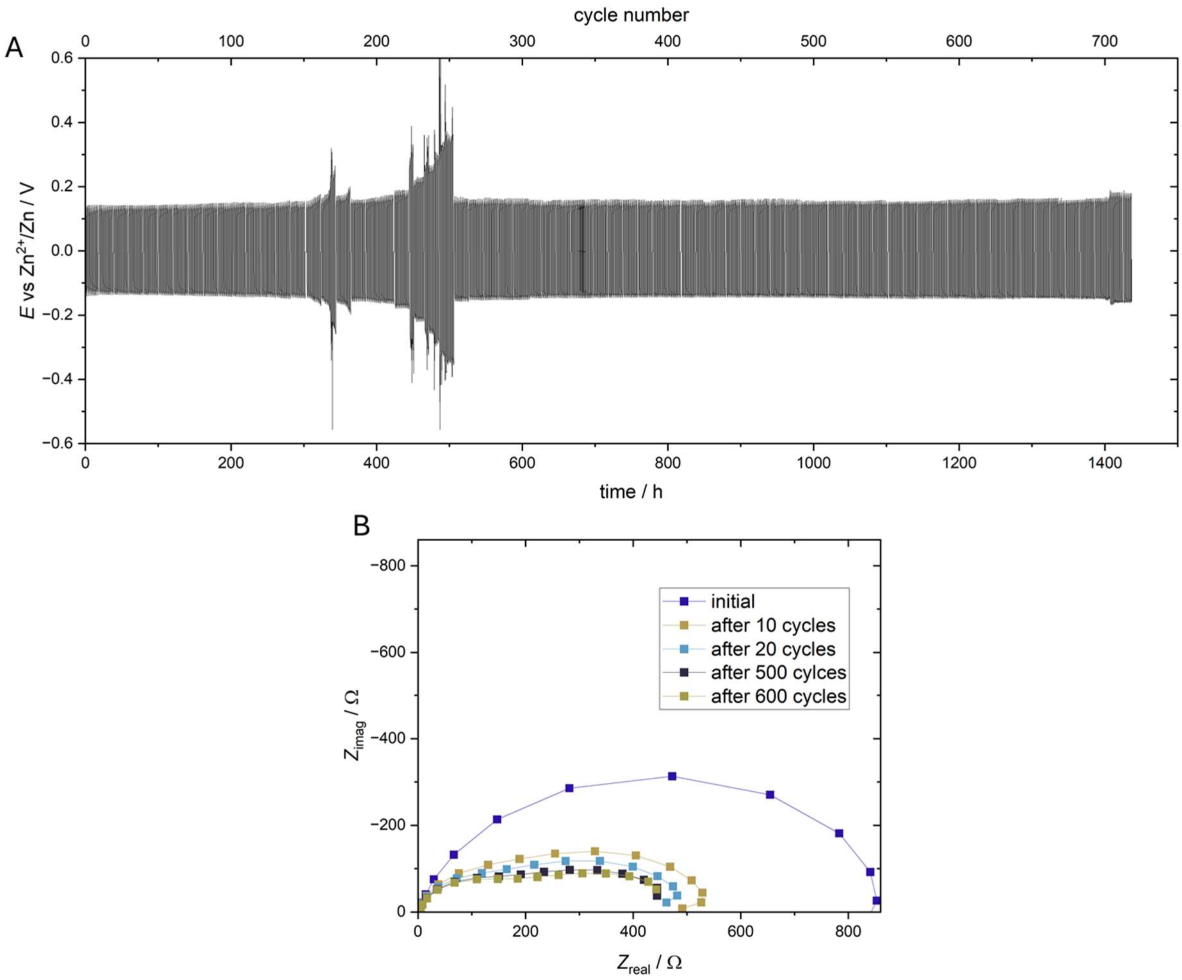

Figure S5:A) Cycling test of symmetrical Zn//Zn cells with an additive blend consisting of PEG300 and CDTA at 1 mA/cm^2 and 1 mAh/cm^2, B) Nyquist Plot of the impedance spectra which were measured before the cycling experiment for (A).

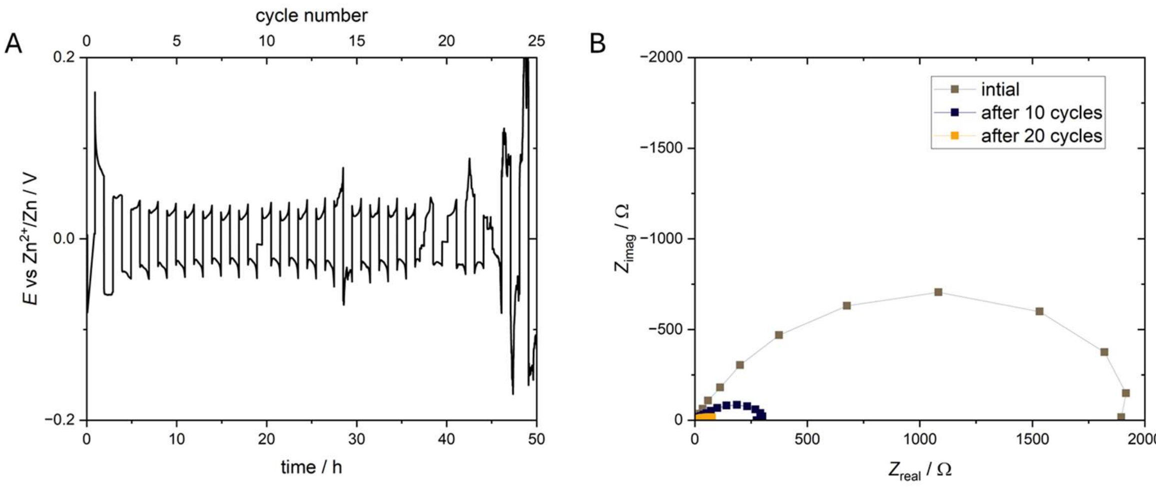

Figure S6: A) Cycling test of symmetrical Zn//Zn cells with an additive blend consisting of EDTA and CDTA at 1 mA/cm^2 and 1 mAh/cm^2, B) Nyquist Plot of the impedance spectra which were measured before the cycling experiment for (A).

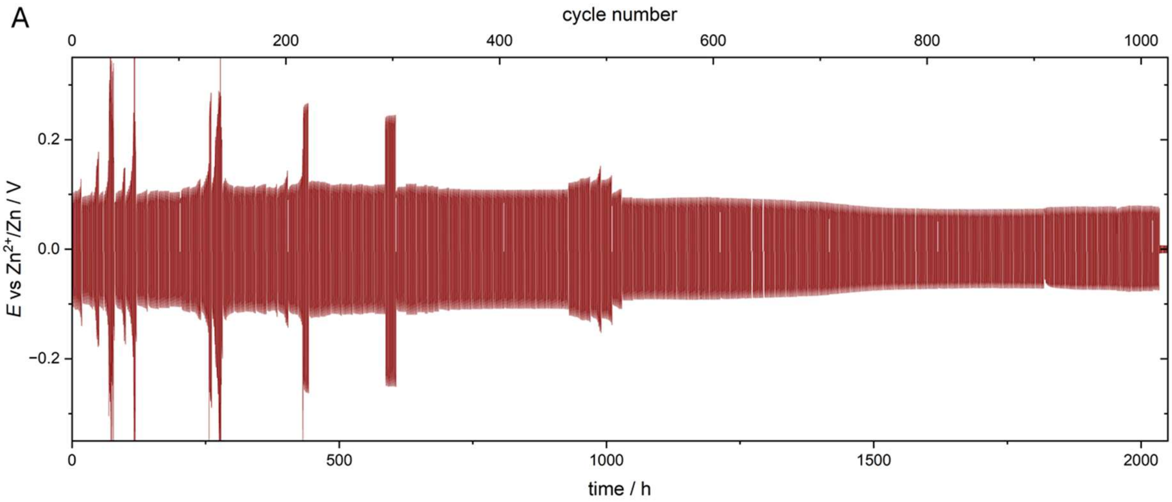

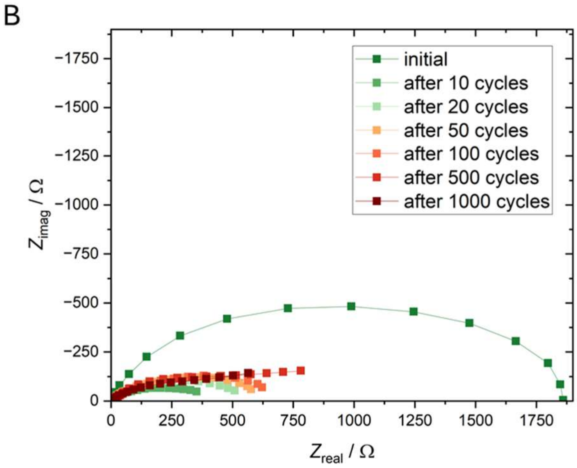

Figure S7: A) Cycling test of symmetrical Zn//Zn cells with an additive blend consisting of SDBS and $Zn(OAc)_2$ at 1 mA/cm^2 and 1 mAh/cm^2, B) Nyquist Plot of the impedance spectra which were measured before the cycling experiment for (A).

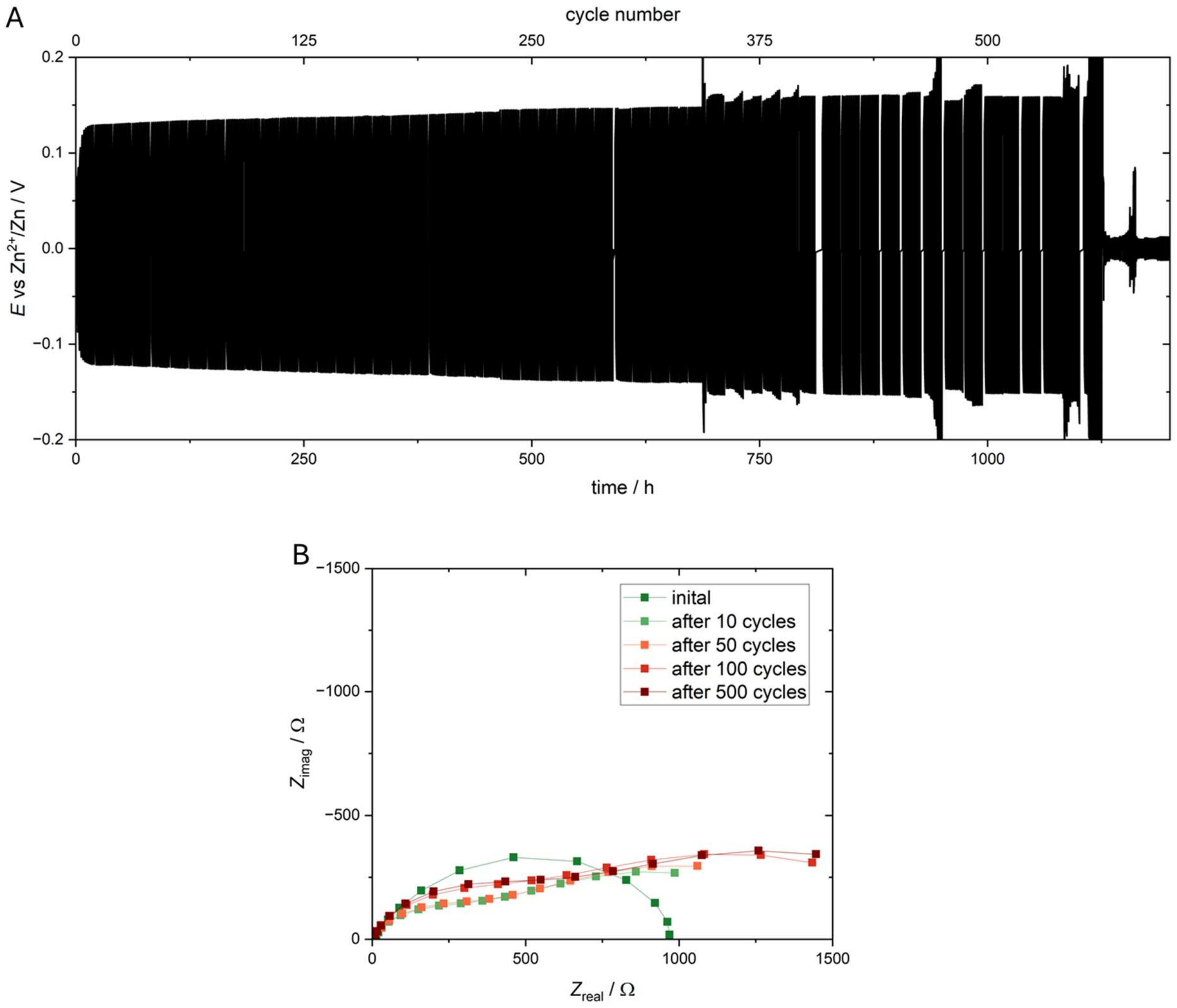

Figure S8: Cycling test of symmetrical Zn//Zn cells with an additive blend consisting of SDBS and L-histidine at 1 mA/cm^2 and 1 mAh/cm^2, B) Nyquist Plot of the impedance spectra which were measured before the cycling experiment for (A).

Table S1:Values calculated from the Tafel plots, contact angles, and the maximum cycle number reached for surfactant-type additives.

	SDBS+EDTA	SDBS+CDTA	PEG, SBDS	PEG+EDTA	PEG+CDTA
Cycle number	1950	550	305	2600	710
I_{corr} / mA/cm^2	0.0010	0.0094	0.0040	0.0116	0.0070
E_{corr} vs Zn^{2+}/Zn / V	0.002	0.021	0.003	0.011	0.014
Coverage	99%	94%	97%	92%	95%
Contact angle	16.9°	36.9°	23.3°	26.0°	39.3°

Table S2: Values calculated from the Tafel plots, contact angles, complex constant of the chelating agent, and the maximum cycle number reached for chelating additives.

	SDBS+EDTA	SDBS+CDTA	EDTA	SDBS+His	SDBS+ZnOAc
Cycle number	1950	550	42	541	710
I_{corr} / mA/cm^2	0.0010	0.0094	0.0094	0.0154	0.0015
E_{corr} vs Zn^{2+}/Zn / V	0.002	0.021	0.021	0.033	0.000
Coverage	99%	94%	90%	99%	99%
Contact angle	16.9°	36.9°	59.1°	25.3°	22.3°
Complex constant $\log(K_{Zn})$	16.4 (EDTA)[16]	17.1 (CDTA)[16]	16.4 (EDTA)[16]	12.3 (His)[17]	1.5 (OAc)[18]

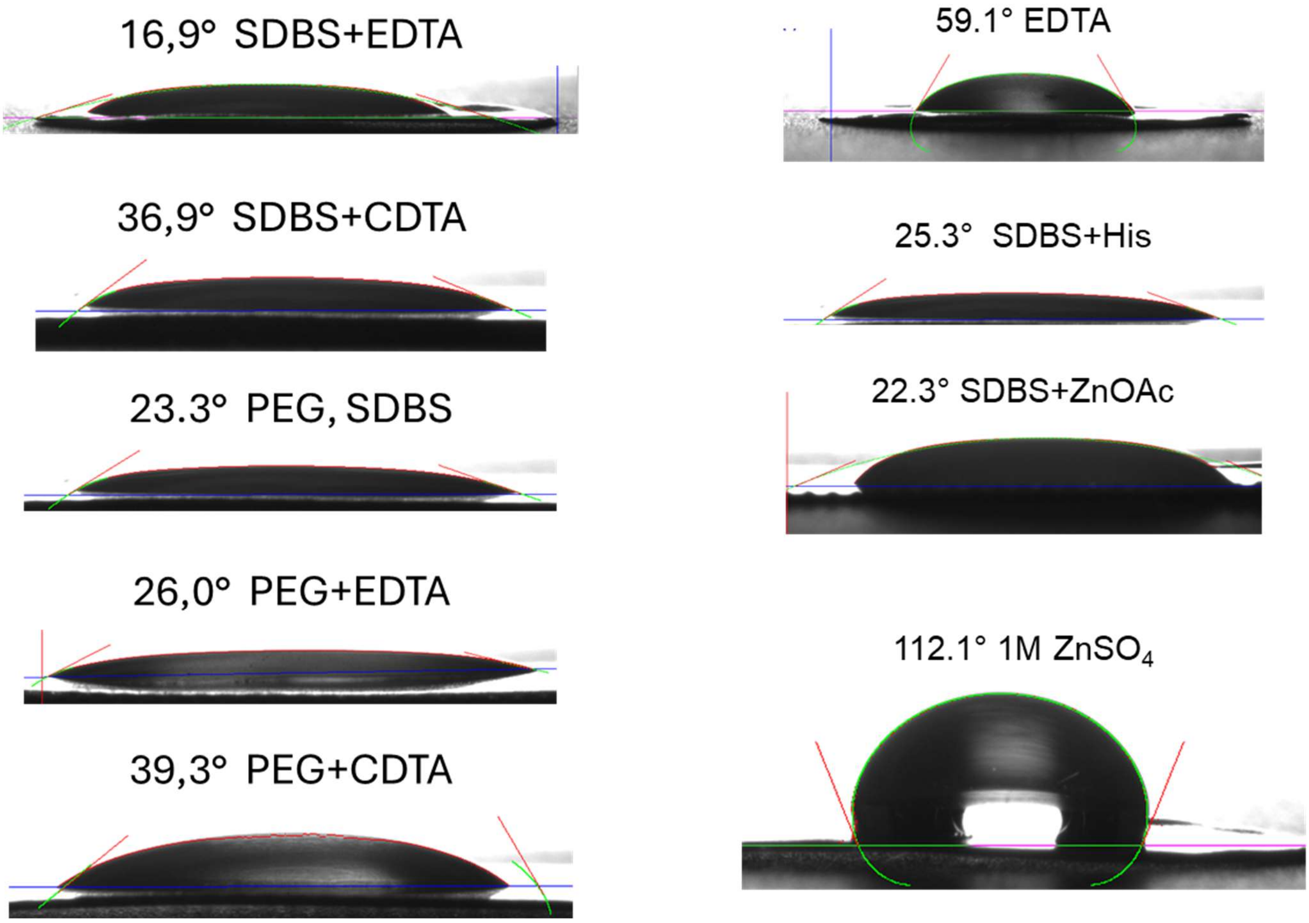

Figure S9: Contact angle measurements of Additive blends on pristine zinc surfaces,

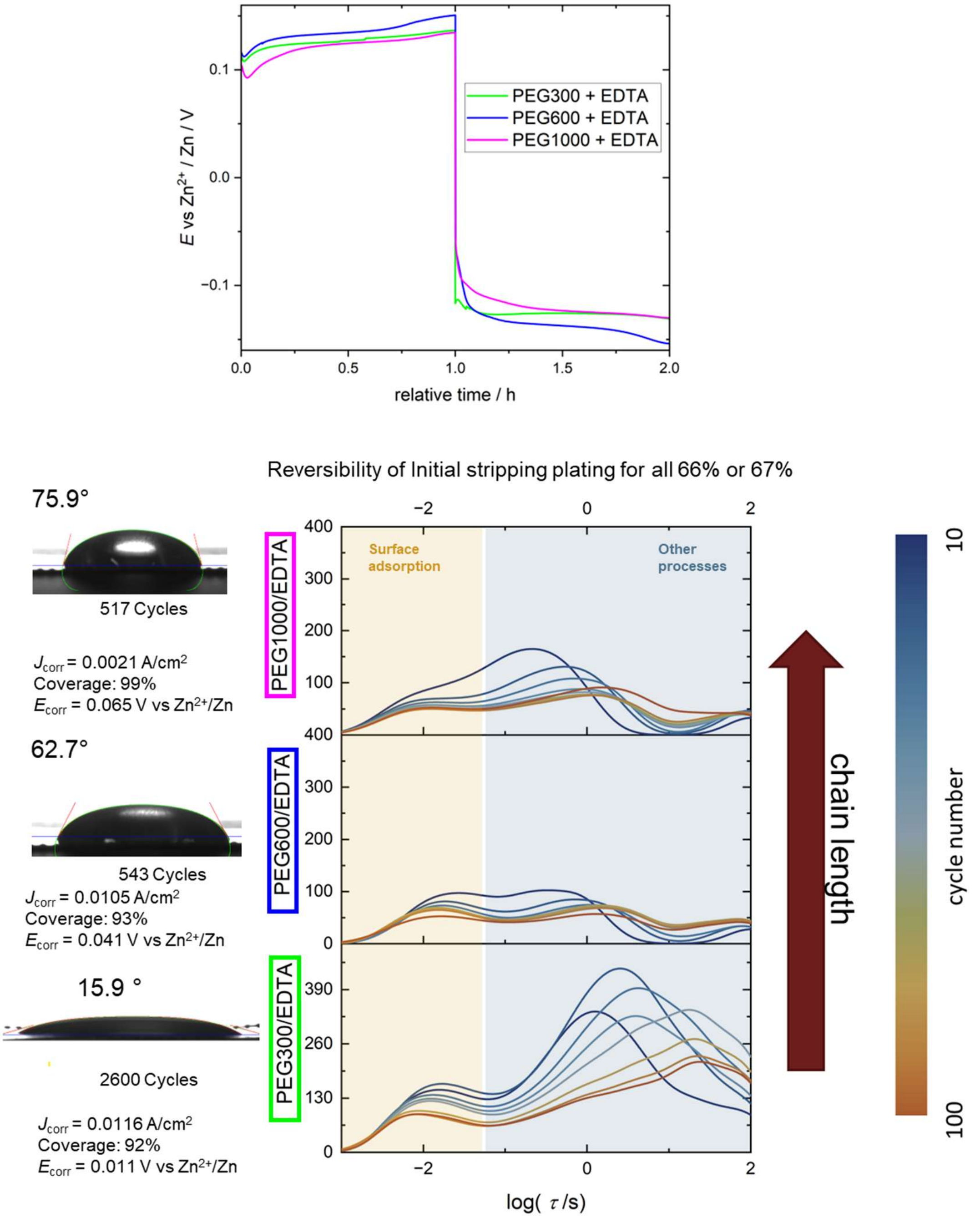

Figure S10: A representative cycle per electrolyte composition is examined from the cycling experiments, DRT plots of the impedance spectra were measured after every 10th cycle for different additive formulations containing PEG in different chain lengths and EDTA. These experiments involved symmetrical Zn//Zn cells with various additive blends containing surface-active additives at 1 mA/cm^2 and 1 mAh/cm. Additionally, contact angle measurements on pristine zinc surfaces, values calculated from the Tafel plots, and the maximum cycle number reached are included.

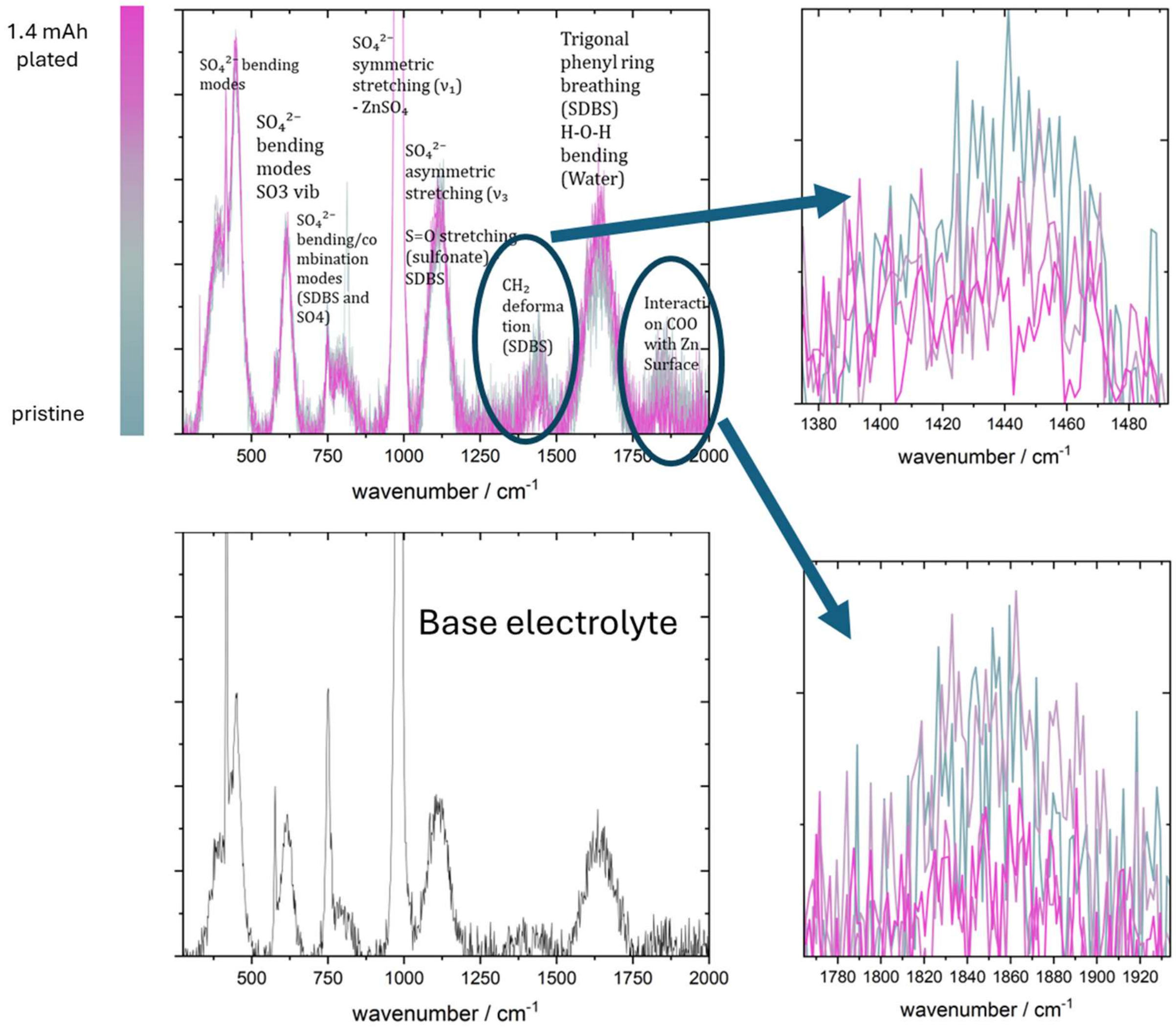

Figure S11: Operando Raman during plating at 0.7 mAh/cm^2 in operando Raman cell by rhd instruments, including assigned peaks, Enlarged view of peak areas, and Raman spectra of base electrolyte.

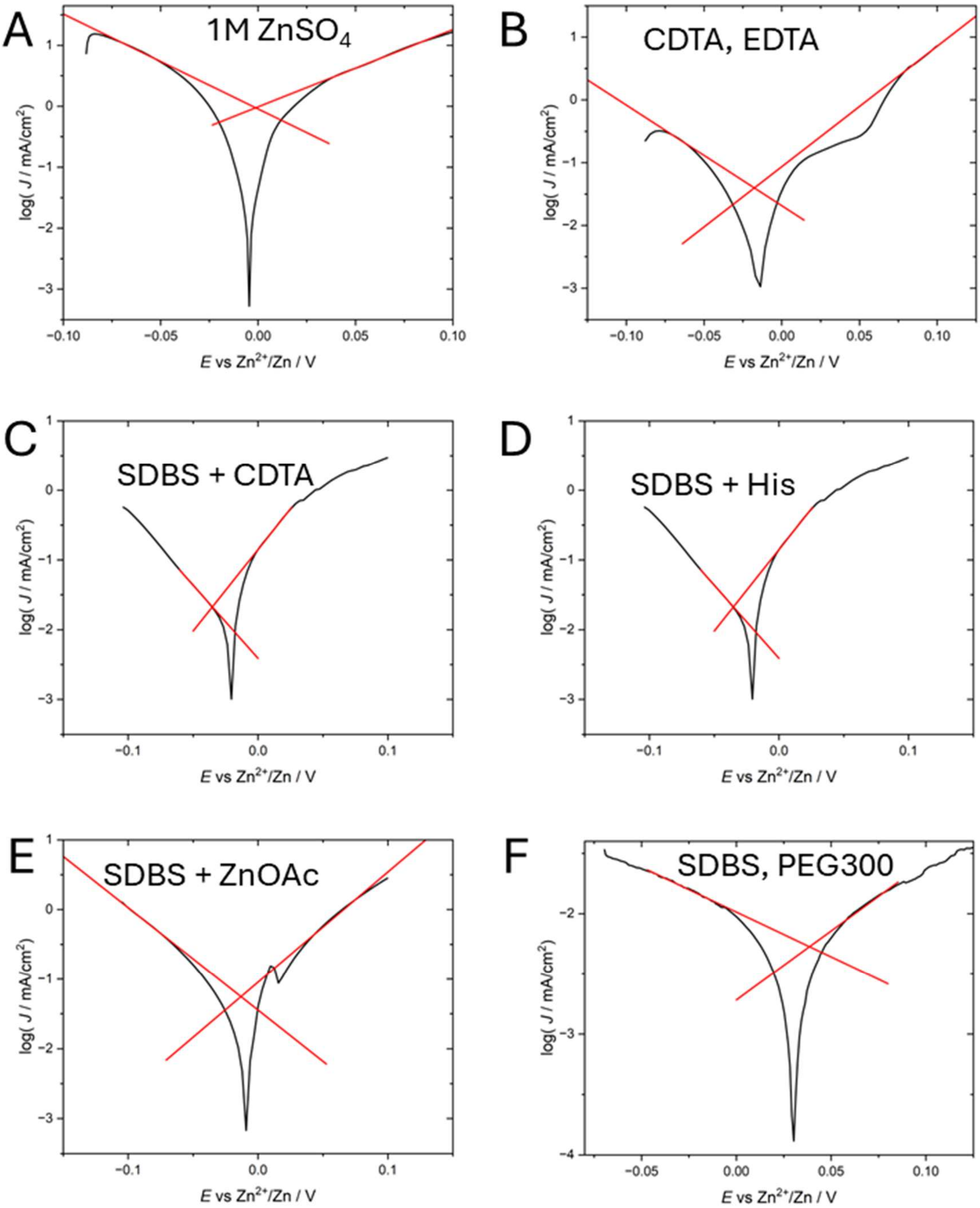

Figure S12: Tafel plots derived from linear sweep voltammetry (LSV) measurements with different electrolytes measured in symmetrical Zn//Zn cells with Zn wire pseudo-reference electrodes and including fitted curves for the determination of the corrosion current and voltage.

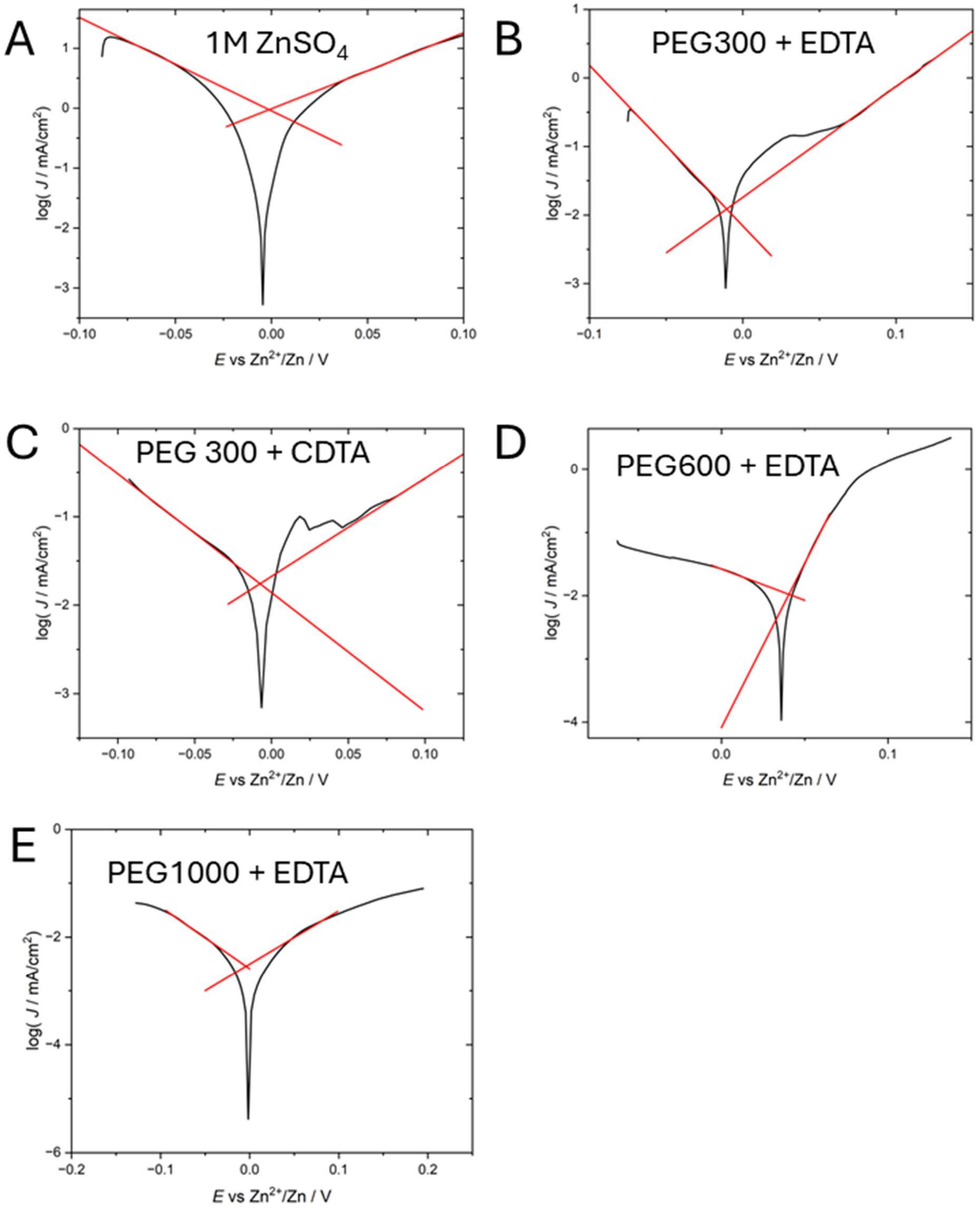

Figure S13: Tafel plots derived from linear sweep voltammetry (LSV) measurements with different electrolytes measured in symmetrical Zn//Zn cells with Zn wire pseudo-reference electrodes and including fitted curves for the determination of the corrosion current and voltage.

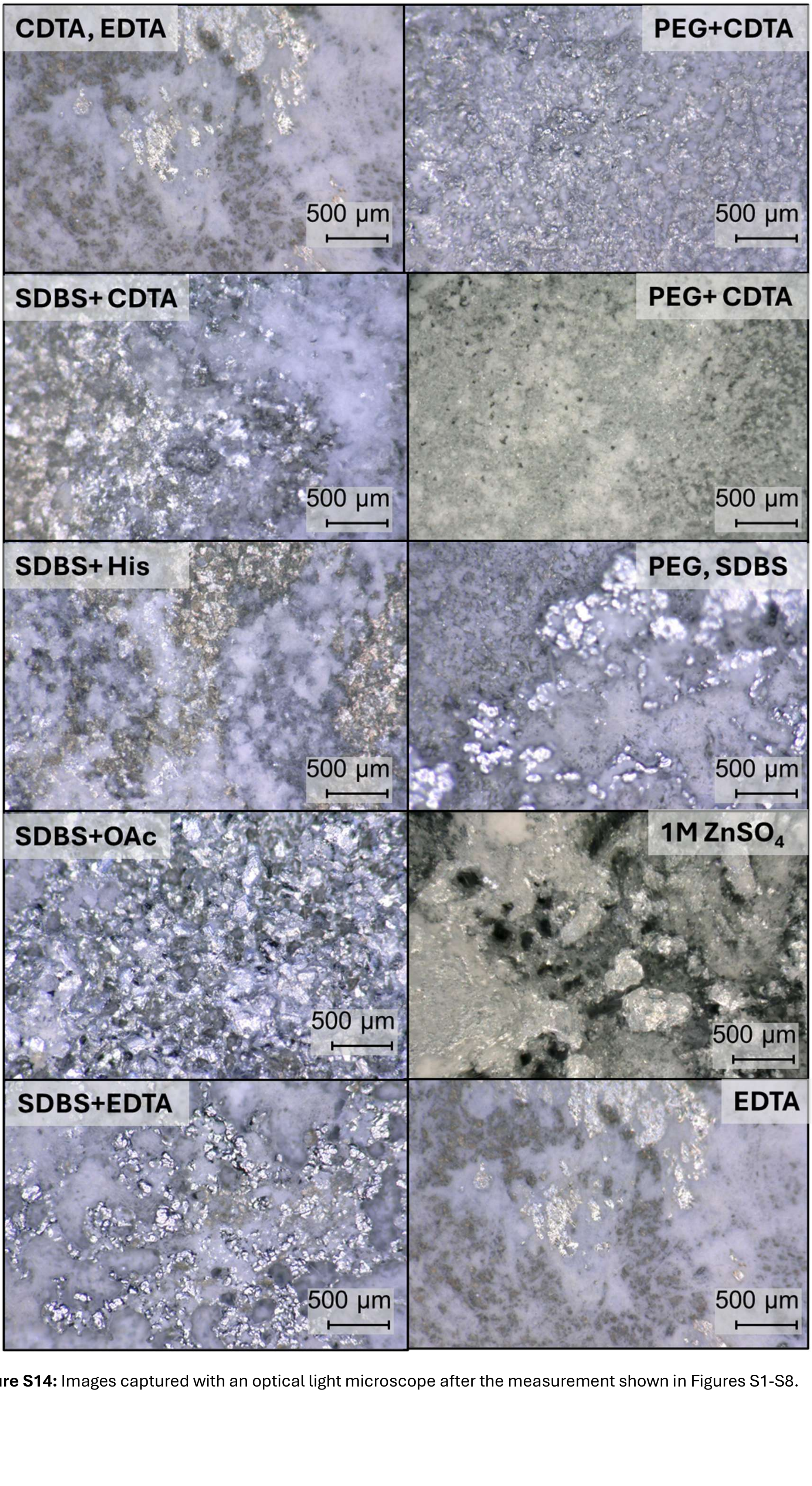

Figure S14: Images captured with an optical light microscope after the measurement shown in Figures S1-S8.

Equation for Surface coverage:

$$\text{surface coverage} = \frac{I_{\text{corr, base electrolyte}} - I_{\text{corr, additive}}}{I_{\text{corr, base electrolyte}}} \qquad (1)^{[19]}$$

with:

- $I_{\text{corr, base electrolyte}}$: corrosion current from LSV measurements and Tafel plots of 1M $ZnSO_4$
- $I_{\text{corr, additive}}$: corrosion current from LSV measurements and Tafel plots of additive-containing cells

Table S3: From DFT calculation derived solvation energies and enthalpies.

Solvation energy	ΔE /kJ/mol	ΔG / kJ/mol
**Zn^{2+}**	-1306.4	-1306.4
**Zn^{0}**	-0.5	-0.5
**Na^{+}**	-302.1	-302.1
**$SDBS^{-}$**	-270.1	-269.1
**SDBS-H**	-45.6	-48.1
**$EDTA^{4-}$**	-2372.0	-2360.2
**$CDTA^{4-}$**	-2413.1	-2396.8

References:

[1] A. D. Becke, *J. Chem. Phys.* **1993**, *98*, 5648–5652.

[2] C. Lee, W. Yang, R. G. Parr, *Phys. Rev. B* **1988**, *37*, 785–789.

[3] S. H. Vosko, L. Wilk, M. Nusair, *Can. J. Phys.* **1980**, *58*, 1200–1211.

[4] P. J. Stephens, F. J. Devlin, C. F. Chabalowski, M. J. Frisch, *J. Phys. Chem.* **1994**, *98*, 11623–11627.

[5] F. Weigend, R. Ahlrichs, *Phys. Chem. Chem. Phys.* **2005**, *7*, 3297.

[6] F. Weigend, *Phys. Chem. Chem. Phys.* **2006**, *8*, 1057.

[7] S. Grimme, J. Antony, S. Ehrlich, H. Krieg, *J. Chem. Phys.* **2010**, *132*, 154104–154123.

[8] S. Grimme, S. Ehrlich, L. Goerigk, *J. Comput. Chem.* **2011**, *32*, 1456–1465.

[9] A. V. Marenich, C. J. Cramer, D. G. Truhlar, *J. Phys. Chem. B* **2009**, *113*, 6378–6396.

[10] F. Neese, *WIREs Comput. Mol. Sci.* **2025**, *15*, e70019.

[11] F. Neese, *J. Comput. Chem.* **2003**, *24*, 1740–1747.

[12] F. Neese, F. Wennmohs, A. Hansen, U. Becker, *Chem. Phys.* **2009**, *356*, 98–109.

[13] D. Bykov, T. Petrenko, R. Izsák, S. Kossmann, U. Becker, E. Valeev, F. Neese, *Mol. Phys.* **2015**, *113*, 1961–1977.

[14] B. Helmich-Paris, B. de Souza, F. Neese, R. Izsák, *J. Chem. Phys.* **2021**, *155*, 104109.

[15] F. Neese, *J. Comput. Chem.* **2023**, *44*, 381–396.

[16] M. E. McMahon, R. J. Santucci, J. R. Scully, *RSC Adv.* **2019**, *9*, 19905–19916.

[17] F. Oakley, N. M. Horn, A. L. Thomas, *J. Physiol.* **2004**, *561*, 525–534.

[18] M. M. Yang, D. A. Crerar, D. E. Irish, *Geochim. Cosmochim. Acta* **1989**, *53*, 319–326.

[19] A. A. Al-Amiery, A. B. Mohamad, A. A. H. Kadhum, L. M. Shaker, W. N. R. W. Isahak, M. S. Takriff, *Sci. Rep.* **2022**, *12*, 4705.
